# The Functional Fitness MOT Test Battery for Older Adults: Protocol for a Mixed-Method Feasibility Study

**DOI:** 10.2196/resprot.5682

**Published:** 2016-06-20

**Authors:** Lex D de Jong, Andy Peters, Julie Hooper, Nina Chalmers, Claire Henderson, Robert ME Laventure, Dawn A Skelton

**Affiliations:** ^1^ Glasgow Caledonian University Institute for Applied Health Research School of Health and Life Sciences Glasgow United Kingdom; ^2^ NHS Lothian Delivering Better Care Hub Western General Hospital Edinburgh United Kingdom; ^3^ Slateford Physiotherapy Clinic Slateford Medical Centre Edinburgh United Kingdom; ^4^ Sighthill Physiotherapy Clinic Sighthill Medical Centre Edinburgh United Kingdom; ^5^ Loughborough University British Heart Foundation National Centre for Physical Activity and Health Loughborough United Kingdom

**Keywords:** physical activity, physical fitness, physical therapists, aged, health behavior, health services for the aged, feasibility studies

## Abstract

**Background:**

Increasing physical activity (PA) brings many health benefits, but engaging people in higher levels of PA after their 60s is not straightforward. The Functional Fitness MOT (FFMOT) is a new approach which aims to raise awareness about the importance of components of fitness (strength, balance, flexibility), highlight benefits of PA, engages older people in health behavior change discussions, and directs them to local activity resources. This battery of tests combined with a brief motivational interview has not been tested in terms of feasibility or effectiveness.

**Objective:**

To assess whether the FFMOT, provided in a health care setting, is appealing to older patients of a community physiotherapy service and to understand the views and perceptions of the older people undergoing the FFMOT regarding the intervention, as well as the views of the physiotherapy staff delivering the intervention. Secondary aims are to assess the feasibility of carrying out a phase 2 pilot randomized controlled trial of the FFMOT, in the context of a community physiotherapy service, by establishing whether enough patients can be recruited and retained in the study, and enough outcome data can be generated.

**Methods:**

A mixed-methods feasibility study will be conducted in two physiotherapy outpatient clinics in the United Kingdom. A total of 30 physically inactive, medically stable older adults over the age of 60 will be provided with an individual FFMOT, comprising a set of six standardized, validated, age-appropriate tests aimed at raising awareness of the different components of fitness. The results of these tests will be used to provide the participants with feedback on performance in comparison to sex and age-referenced norms. This will be followed by tailored advice on how to become more active and improve fitness, including advice on local opportunities to be more active. Subsequently, participants will be invited to attend a focus group to discuss barriers and motivators to being more active, health behavior change, and the scope for individuals to improve their PA levels. To inform the design of a future trial, descriptive (eg, recruitment and retention rates), quantitative (Community Healthy Activities Model Program for Seniors; CHAMPS physical activity questionnaire), and qualitative data (focus group discussions, semi-structured staff interviews) will be collected.

**Results:**

Recruitment and enrolment for the trial started in September 2015. Follow-up will be completed in June 2016. Results are expected to be available at the end of 2016.

**Discussion:**

Allied health professionals play a key role in encouraging older adults to increase their PA, but with little evidence on how best to do this within their clinical practice. The purpose of this feasibility study is to examine the introduction of a new service: The FFMOT. The views and perceptions of the older people undergoing the FFMOT and relating to its delivery in clinical practice will be explored. Data, which will inform the feasibility of a randomized controlled trial of effectiveness of the FFMOT in promoting improved PA, will be reported.

**Trial Registration:**

International Standard Randomized Controlled Trial Number (ISRCTN): ISRCTN38950042; http://www.isrctn.com/ISRCTN38950042

## Introduction

Increasing physical activity (PA) brings many health benefits, improved health care outcomes, and reduced health care costs [1]. PA recommendations for the health of older adults include being active daily and doing activities that improve strength and balance at least twice a week. Over a week, activity should add up to at least 150 minutes of moderate intensity activity in bouts of 10 minutes or more [1]. However, only one in five people aged 65-74 years and one in ten over-75 years achieve recommended activity levels [2].

The Scottish Government has recommended that allied health professionals play a central role in encouraging people to be more active [3]. Physiotherapists are expected to raise the question of activity levels at each initial consultation, and promote PA at any given opportunity to improve the overall health and well-being of people using their services. However, engaging older people in higher levels of PA is not straightforward. In fact, evidence for the effectiveness of interventions to promote healthier levels of PA among adults [4-6] and older adults [7] is not compelling. Recently, one study showed that older people recruited through general practice and undergoing a 6-month group exercise intervention, increased their moderate intensity activity by about 15 minutes a day a year after the intervention finished [8]. However, this approach is costly and many older people do not find group exercise appealing. Therefore, not only effective but cost-effective approaches to increase PA are needed.

Rikli and Jones [9] previously developed and validated a battery of performance tests to provide a means of assessing key physiological parameters that are associated with functional mobility in independent older adults between 60-90 years. This test battery was recently adapted and further developed into the Functional Fitness MOT (FFMOT) [10]. The FFMOT is a new approach that aims to raise awareness of the importance of components of fitness such as strength, balance, and flexibility among the over-60s, highlight the benefits of PA, engage older people in health behavior change discussions, and direct them to appropriate local activity resources. It involves one face-to-face session to measure participants’ fitness using seven age-appropriate functional fitness tests. Immediate feedback is given on the person’s abilities in each test in relation to normal values for their age and sex, followed by tailored advice on how to become more active and improve fitness. This brief motivational set of tests and discussion is based on the principles of health behavior change, incorporating awareness raising, breaking down barriers, improving self-efficacy, building on history of activity, incorporating needs and preferences, and goal setting [11]. To date the FFMOT has been piloted in non-clinical settings only [10].

Due to the lack of in-depth research and existing data about the FFMOT in a clinical setting, a feasibility study is indicated [12]. Therefore, in line with Medical Research Council guidance [13], the first phase of this work program is a feasibility study to evaluate the approach in clinical settings. According to the guidelines for the design of feasibility studies proposed by Bowen et al [12], the key areas of focus for this study are (1) *acceptability, demand, implementation, and practicality* for the participants, that is, to assess whether the FFMOT, provided in a health care setting, is appealing to older patients of a community physiotherapy service, and to understand their views and perceptions undergoing the FFMOT regarding the intervention, and (2) *acceptability, demand, implementation, practicality, and integration* for the staff delivering the FFMOT, that is, to assess their views on delivering the intervention to the target participants and within the setting of a community physiotherapy clinic. Secondary aims are to assess *limited efficacy*, that is, the feasibility of carrying out a phase 2 pilot randomized controlled trial of the FFMOT, in the context of a community physiotherapy service, by establishing whether enough patients can be recruited and retained in the study, and relevant outcome data can be generated.

## Methods

### Design

This is a mixed-method phase I feasibility study with a pre-post design.

### Participants

A convenience sample will be identified and recruited from one UK health board area, from among the current caseloads of community physiotherapists working in two physiotherapy clinics for musculoskeletal (MSK) conditions. Participants are eligible for participation if they are (1) aged 60 years or above, (2) not physically active for at least 30 minutes in 5 days or more, or for at least 150 minutes (2½ h) in total in the past week, as indicated by the questions on the Scottish Physical Activity Screening Question [14], and (3) interested in increasing their level of PA (where this is seen as an appropriate goal by the screening physiotherapist). Participants are excluded if the screening physiotherapist identifies health risks (contraindications to exercise; eg, cardiovascular disease) which prevents participation, and if they have been diagnosed with moderate/severe cognitive impairment, a learning disability, severe mental illness, or the screening physiotherapist believes that any of these impairments/disorders are present.

Where patients are deemed eligible, the screening physiotherapist will introduce the nature of the study verbally to them. The screening physiotherapist will then ask potential participants if they wish to receive a study information pack and, if so, they will be given the pack during the appointment. Participants who are interested will then complete a contact details form and post it to the main researcher, who will subsequently telephone them in order to explain the consent procedures, and make an appointment for them to attend an FFMOT session. All participants will continue to receive standard physiotherapy assessment and intervention as deemed appropriate by the screening physiotherapist, irrespective of their involvement in the study.

### Sample Size

This study is a feasibility study so no formal power calculation has been carried out. However, rates of participant retention at 3-month follow-up in feasibility studies of exercise interventions for adults range between 71-100% [15-18]. Adopting a conservative estimate of 70% retention, 30 participants will be recruited with the aim of retaining 21 at the 3-month follow-up. Six blocks of FFMOT sessions will be provided over 12 weeks, with provision to run two to three additional blocks beyond this if required to achieve the recruitment target.

### Physiotherapy Staff

All physiotherapists working at the two participating physiotherapy clinics, who assess and treat MSK outpatients, will be eligible to screen patients as potential participants.There are 3 physiotherapists involved in coordinating the study within the service. They will also supervise the 2 trained technical instructors (TIs) who will deliver the FFMOT. The TIs will receive training in both the delivery of the FFMOT [19] and in motivation and support strategies for engaging older adults in PA [20].

### Functional Fitness MOT

Each recruited participant will be provided with an individual FFMOT session, which lasts for 45-60 minutes. The FFMOT in this study comprises 6 of the 7 standardized, validated, age-appropriate tests aimed at raising awareness of the different components of fitness:

1) 30 Second Chair Stand [21,22], as an indicator of lower limb strength and endurance;

2) Chair Sit and Reach [21,23,24], as an indicator of lower limb flexibility;

3) Back Scratch [21], as an indicator of upper body shoulder flexibility;

4) 8 Foot Up and Go [21,25], as an indicator of dynamic balance;

5) Handgrip Strength [26], as an indicator of strength;

6) Single Leg Stance [27,28], as an indicator of static balance.

The 6-minute walk test, for endurance, will not be used within this FFMOT battery due to space constraints within the clinic.

The results of these tests will be used to discuss the different components of fitness, to highlight the individual’s strengths and weaknesses in these components, and to provide the participants with personal feedback on performance in comparison to sex and age-referenced norms. This then allows discussion around the person’s activity history, needs, and preferences so that information about local opportunities to engage in PA can be introduced and encouraged. Specific goal setting (short and longer term) is discussed and each participant is provided with information about appropriate local activity opportunities and home exercises, based on their FFMOT results. This information will be provided in the form of written booklets/leaflets and links to websites of some of the opportunities available. For those who wish to exercise at home, home exercise booklets will be provided [29].

### Data Collection

#### Descriptive Data

The following information from every patient screened during the recruitment phase will be collected: (1) the clinic at which the patient was assessed/treated, (2) sex, (3) age, (4) postcode, (5) patient responses to the 3 questions in the Scottish Physical Activity Screening Question, (6) eligibility criteria, (7) whether the study was introduced verbally to the patient, and (8) whether the patient accepted the study information pack. Recruitment and retention rates at the key stages of the study (eg, attendance for the FFMOT and focus group, return of completed follow-up questionnaires) will also be monitored and recorded.

The Community Healthy Activities Model Program for Seniors (CHAMPS) Activities Questionnaire for Older Adults [30] will be administered before the FFMOT. This questionnaire is a valid and reliable instrument to measure recent levels and types of PA in detail in this age group. Twelve weeks after the FFMOT session a follow-up CHAMPS, and a bespoke post-intervention questionnaire will be sent by post. The latter questionnaire aims to measure the participants’ contact with community organizations and facilities with a PA focus since attending the FFMOT session.

#### Qualitative Data

Between 2-4 weeks after participants have attended the FFMOT session, they will be invited to attend a 60-90 minute focus group. Each group will consist of a maximum of 12 participants. The focus groups will be moderated by the first author (LDdJ) according to a bespoke, predesigned discussion guide. The discussion guide contains a set of semi-structured, open-ended questions. The interview process during the focus group comprises a 4-step approach to ask the participants the following:

1. Their reason(s) for taking part in the study.

2. Their PA awareness (eg, reasons for not adhering to PA guidelines, perception changes after the FFMOT, views on and awareness about the importance and benefits of PA, perceived importance of and changes in PA behavior since the FFMOT, and changes in awareness of local opportunities to become more physically active).

3. The FFMOT and its appeal (eg, how it felt to undergo it, what was liked and disliked, clarity of the feedback by the TIs, impact of the test results when compared with other people of the same age group, reasons for (not) recommending it to others, suggestions for improvement to make it more appealing, and views on timing and location of provision of the service).

4. Their experiences of the study procedures (eg, concerns about taking part, clarity of the participant information sheet, experiences with completing the consent procedure and CHAMPS questionnaires).

Four semi-structured individual interviews, of up to 60 minutes, with the supervising physiotherapists and the 2 TIs will be held no more than 2 weeks after all FFMOT sessions have been provided. The interview process for these interviews comprise a 3-step approach to ask the staff about the following:

1. Previous experiences in working with older people, therapy, fitness testing, exercise, research, and background knowledge of fitness tests/testing.

2. Perceptions of the FFMOT (eg, views on the pretrial training, appropriateness and constraints of the screening criteria and fitness tests, experiences with administering the tests, representativeness of the participants’ results, issues during administration of the tests, views on the appeal, benefits, disadvantages, and value of the FFMOT, suggestions for improvement).

3. The FFMOT in the context of a community physiotherapy service (eg, appropriateness of the physiotherapy service to deliver the FFMOT, views on feasibility, barriers and motivators to deliver it in a physiotherapy service, views on improvements in the delivery of the FFMOT).

All focus group discussions and staff interviews will be audio recorded onto an encrypted digital recorder.

### Analysis

As this is a feasibility study, the analyses will be primarily descriptive. Reasons for exclusion, participant recruitment and retention rates, number of completed questionnaires returned, and incidence of missing questionnaire item responses will be explored. Additional analyses will be conducted of any demographic differences (including sex, age, the clinic where screening occurred, the Scottish Index of Multiple Deprivation [31] rank of participant’s home postcode, and travelling distance from home to clinic between (a) eligible and non-eligible patients, (b) eligible patients who accepted an information pack and those who did not, (c) eligible patients who were recruited and those who were not, and (d) completers and non-completers. CHAMPS data will be compared between baseline and 12-week follow-up to provide an initial estimate of effect size to inform the design of a future trial. A previous study [8] using CHAMPS has shown positively skewed distributions, so it is likely that similar logarithmic transformations (CHAMPS score +1) will have to be carried out. Qualitative data (focus group and staff interview audio recordings) will be fully transcribed and coded using thematic analysis. Thematic analysis will be carried out by two of the authors (LDdJ, DAS), with all authors agreeing the final themes.

### Informed Consent

Written informed consent will be obtained from all participants. The decision regarding participation in the study is entirely voluntary. The researcher will emphasize to potential participants that consent regarding study participation can be withdrawn at any time without giving any reason, and without affecting their physiotherapy/medical care. It will also be emphasized to potential participants that all of the information that is collected about them during this project (personal, medical, and audio recordings) will be anonymized, will not affect their confidentiality in any way, and that it will not be possible to identify information about them when the results of the study are published.

### Ethical and Organizational Review

A favorable ethical opinion of this study was granted by the NHS South East Scotland Research Ethics Committee 01, Scotland UK (Reference 15/SS/0118). Approval was also granted by the NHS Lothian Research & Development Office (Reference 2015/0283). The study is sponsored by Glasgow Caledonian University (Reference RIE13-127).

## Results

Recruitment and enrolment for the trial started in September 2015. Follow-up will be completed in June 2016. Results are expected to be available at the end of 2016.

## Discussion

Allied health professionals are tasked with playing a key role in encouraging older adults to increase their PA. Older adults need unique consideration in how they are recommended to become more physically active, in particular doing activities that improve strength and balance, but there is little evidence for allied health professionals on how best to do this within their clinical practice. The FFMOT is a new approach that aims to raise awareness among the over-60s of the importance of components of fitness such as strength, balance and flexibility, highlight the benefits of PA, engage older people in health behavior change discussions, and direct them to appropriate local activity resources. However, there is no existing data about whether the FFMOT could work in this setting, and therefore performing a feasibility study is indicated. The purpose of this mixed-method phase I feasibility study with a pre-post design is to examine the introduction of the FFMOT for older adults, within two community physiotherapy MSK clinics in the United Kingdom. Specifically, the views and perceptions of a convenience sample of older people undergoing the FFMOT relating to the delivery of the FFMOT in clinical practice will be explored. Patient recruitment/retention rates, and the extent to which outcome data can be collected, will be evaluated with a view of planning a future randomized controlled trial of the FFMOT compared with the usual treatment provided (recommendations for increased activity). Participants included in this study will be provided with single, 45-60 minute consultation, including age-appropriate functional tests, which could potentially increase their motivation to become more physically active. Increased PA could help them improve their gait, balance, mobility, and general health in the longer term. If this feasibility study suggests the FFMOT can work in the clinical setting, then a definitive RCT will be proposed in order to look at whether the FFMOT is efficacious and effective at increasing PA in older adults.

## Abbreviations

FFMOT: Functional Fitness MOT (Note: in the UK the abbreviation MOT refers to the annual test of vehicle safety and as such is generally recognized by the population. In the same vein, the Functional Fitness MOT is a battery of human physical fitness tests so participants recognize the term)
MSK: musculoskeletal
PA: physical activity
TI: technical instructor
